# Nurses’ self-regulation after engaging in end-of-life conversations with advanced cancer patients: a qualitative study

**DOI:** 10.1186/s12912-024-02016-6

**Published:** 2024-05-22

**Authors:** Jiayi Du, Zifen An, Chunyu Wang, Liping Yu

**Affiliations:** 1 Radiation Oncology Center, Chongqing University Cancer Hospital, No. 181, Han Yu Road, Shapingba District, Chongqing, 400030 China; 2 Wuhan University School of Nursing, No.115, Donghu Road, Wuchang District, Wuhan, Hubei Province 430071 China

**Keywords:** End-of-life conversation, Cancer, Nurses, Self-regulation, Descriptive research, Heart to Heart Cards Game

## Abstract

**Background:**

Self-regulation is crucial for nurses who engage in in-depth end-of-life conversations with advanced cancer patients, especially in cultural contexts featuring death taboos. An improved understanding of the self-regulation process of nurses can help them address negative emotions and promote self-growth more effectively. Therefore, this study aimed to explore nurses’ self-regulation process after end-of-life conversations with advanced cancer patients.

**Methods:**

This study employed a descriptive, qualitative approach. Seventeen nurses from four hospitals and a hospice unit in mainland China were interviewed between September 2022 and June 2023. Data were collected through face-to-face semistructured interviews. A thematic analysis method was used to analyse the data following the guidance of regulatory focus theory.

**Results:**

Three main themes were developed: self-regulation antecedents include personality, experience, and support; promotion or prevention is a possible self-regulation process for nurses; both self-exhaustion and self-growth may be the outcomes of self-regulation, as did seven subthemes. Personality tendencies, life experience, and perceived support may affect nurses’ self-regulation, thereby affecting their self-regulation outcomes.

**Conclusions:**

Nurses exhibit different self-regulatory tendencies and self-regulation outcomes. The provision of peer support and counselling support to nurses is highly important with regard to achieving good self-regulation outcomes.

## Background

 Cancer is a leading cause of mortality, accounting for nearly 10 million annual deaths worldwide, i.e., 1 in 6 deaths [1, 2]. Patients with advanced cancer always face a complex array of concerns and anguished choices, and they thereby require support and guidance from health care personnel [3, 4]. Studies have emphasized the important role of health care providers in end-of-life conversations about patients’ preferences and wishes [5, 6] since these end-of-life conversations have the potential to improve the quality of life and well-being of patients as well as their family members, reduce unwanted and aggressive treatments and enhance patient-centred care [7].

The literature has revealed several barriers to the initiation of end-of-life conversations on the part of health care providers, including human factors, time constraints, and challenges in the work environment [8, 9]. Various tools for clarifying and documenting patients’ wishes have been implemented worldwide to help health care professionals initiate and organize end-of-life conversations [10, 11]. The Heart to Heart Cards Game (HHCG) is a tool developed by the Chinese American Coalition for Compassionate Care based on Go Wish cards under the guidance of cultural appropriateness theory [11], and previous studies have proven that the HHCG can help nurses initiate end-of-life conversations with cancer patients, thereby improving their understanding of patients’ preferences and leading to improvements in patients’ quality of life [12, 13]. Similarly, we cannot ignore the fact that the person who must navigate end-of-life conversations is a health care provider.

Unlike daily conversations focused on events or tasks, end-of-life conversations focus on psychosocial, psychological, and spiritual issues, thereby approaching the patient’s inner self; moreover, discussions of death and dying are inevitable in this context [11–13]. Studies have shown that health care personnel are concerned about causing distress for patients or decreasing their hope, that they feel discomfort when discussing “impolite” or “taboo” topics, and that they lack confidence due to limited training in end-of-life conversations [8, 9]. In end-of-life conversations, we focus on the patient’s health, emotions, and needs, whereas the corresponding impact on nurses after they experience such a difficult conversation is less emphasized.

Research has shown that oncology nurses exhibit a moderate risk of burnout and an extremely high risk of compassion fatigue; these results can be at least partially attributed to their encounters with the dying process [14]. A mixed-method study of oncology nurses [15] revealed that nurses feel overwhelmed when they witness suffering and that they cope with this situation through self-care and spirituality. In addition, in a qualitative study, nurses remarked that the experience of caring for cancer patients changes their mode of thinking [16]. We believe that in-depth end-of-life conversations have a certain impact on nurses and that self-regulation, as the foundation of psychological health, plays an important role in their ability to cope with these conversations.

This study uses regulatory focus theory to shape the interview guide and inform the results. According to regulatory focus theory, two systems determine our tendency and strategy to pursue goals [17]. Individuals who exhibit a dominant promotion system (promoters) focus on obtaining gains, whereas individuals with a dominant prevention system (preventers) focus on avoiding losses [17, 18]; accordingly, if no benefits are gained (promoters) or danger is felt (preventers), negative consequences may emerge [19]. Regulatory focus theory has proven to be useful for understanding self-regulation and has been applied in research on feedback mechanisms and communication effectiveness as well as to the development of interventions [19–22]; therefore, we aim to explore the experiences of and self-regulation exhibited by nurses in the context of in-depth end-of-life conversations with advanced cancer patients based on this theory.

### Aim

This study analyses the experiences of and self-regulation exhibited by nurses in the context of in-depth end-of-life conversations with patients with advanced cancer. The relevant data lay a foundation for future nursing management and education guidance.

## Methods

### Design

We used a qualitative descriptive design [23–25]. This approach is based on naturalistic inquiry principles [23, 26]. We chose this approach since it is especially suitable for obtaining straightforward and largely unbiased answers to questions of particular relevance to practitioners and since it enables researchers to obtain an in depth, rich understanding of nurses’ experiences with end-of-life conversations [23]. We used the Consolidated Criteria for Reporting Qualitative Studies (COREQ) [27] to report the study design, conduct, analysis, and findings.

### Participant recruitment

Purposive sampling and snowball sampling were used to recruit information-rich participants. Given the difficulty of defining the notion of an in-depth end-of-life conversation simply, we consider the HHCG to represent an in-depth end-of-life conversation. The inclusion criteria for the nurses were as follows: experience playing the HHCG with advanced cancer patients during the last three months and willingness to share their experiences. The exclusion criteria were as follows: inability to participate in a face-to-face interview and inadequate ability to speak fluent Mandarin.

The corresponding author contacted two nursing department directors, who provided us with a personnel list and contact information based on our request. We contacted all the individuals on the list by text message or telephone call. For nurses who met the criteria, we further explained the study and invited them to participate in the interview. If the nurse verbally agreed to participate, we scheduled an appointment at the participant’s convenience. To expand the diversity of the sample in terms of age, education level, work experience, and frequency of playing HHCG, we ask all nurses to recommend other potential participants to us. Two of the interviewees recommended other participants to us. Twenty-one nurses were approached, 19 nurses were eligible, and all of these individuals consented to participate in the interview. Recruitment was stopped when data saturation was reached. Data saturation was achieved after 17 nurses had been interviewed.

### Data collection

Data were collected through face-to-face and semistructured interviews with nurses, which were conducted between September 2022 and June 2023 in mainland China. An initial interview guide was designed under the guidance of regulatory focus theory based on a review of the literature concerning the experiences of oncology nurses and hospice nurses conducted by the first author [17–19]. Then, two nurses were selected for a preinterview (the preinterview results were not included in the study analysis), and the formal interview guide was adjusted in accordance with the preinterview results. Following the data analysis, the interview questions became more structured (Table 1).

Quiet interview locations were chosen based on the participants’ preferences, including the head nurse’s offices, the nurses’ lounge, a conference room, and participants’ cars. All interviews were conducted in Chinese by the first author and were audio recorded. The interviewer was a trained female graduate student with qualitative research experience and work experience in the hospice unit and the oncology department. Except for two nurses from the hospice unit, the interviewer did not know the other interviewees. The entire interview process was completed in a natural state, and no other persons were present to ensure that respondents were interviewed with an open mind. The interviews lasted approximately 46 min each (ranging from 28 to 79 min), and four of the nurses participated in two interviews. Field notes were written immediately after each interview, and the interview recordings were transcribed into text within 24 h.

**Table 1:** Interview guide

Main question	
What is your experience with playing Heart to Heart Cards Game with advanced cancer patients?	
What impact do you think that the Heart to Heart Cards Game can have on you?	
How do you regulate yourself during or after the game?	
What factors do you think affect your self-regulation?	
Are you willing to continue playing the Heart to Heart Cards Game with advanced cancer patients and why?	
What support do you think you need if you want to continue playing the Heart to Heart Cards Game with advanced cancer patients?	

### Data analysis

The data collection and data analysis were performed concurrently. A thematic analysis using a deductive/inductive hybrid approach was used to identify nurses’ self-regulation [28, 29]. Considering the issue of language translation, we tried to incorporate the contextual coding approach [30]. We used Chinese for the transcription and preliminary code. First, the two coders read the entire transcript of each participant’s interview multiple times to immerse themselves in the data and obtain a sense of the participants’ experiences as a whole. The coders then independently coded the transcripts line by line by hand to identify possible coding units based on actual words or phrases included in the transcripts. Subsequently, the coders compared and discussed the coding units of the transcripts until consensus was reached. The coders subsequently condensed the coding units based on shared characteristics. Several additional abstract coding units were extracted and clustered into subthemes and main themes based on an iterative and inductive process. Encoding was performed using a combination of manual encoding and NVivo 12 software. The categories and codes drawn from the transcripts were translated into English by three researchers and checked by an English teacher. In addition, in this study, saturation was deemed to have been achieved when the two coders and a qualitative expert noted repetition in the participants’ responses and observed that no new codes related to the nurses’ perspectives on the HHCG developed.

### Ethical considerations

The Medical Ethical Committee of Wuhan University approved this study (2020YF0081). The nurses who participated in the study were informed of the purpose of the study, and oral and written consent was obtained. Permission to use direct quotations from participants was obtained.

### Trustworthiness

We improved the trustworthiness of the study in terms of four aspects: credibility, dependence, conformability and transferability. First, the first author established a trusting relationship with the participants before the interviews. Second, to avoid premature closure of the analysis, we coded separately for each data segment and conducted constant comparative analysis of the data [31]. Besides, we maintained a degree of reflexivity by regularly writing and reviewing a reflective journal and memos to record thoughts, biases, and interpretations about the data, and ensure that any preunderstandings or presumptions could be set aside to the greatest extent possible [31]. Third, any differences in the categories were discussed by three researchers with the goal of reaching a consensus, and the text was then sent to the participants for feedback to ensure the stability of the results. To contextualize a narrative, we noted the participants’ age, gender, and some other relevant demographic information in the quotes [32].

## Results

Participants, including four hospitals and a hospice unit’s nurses (*n* = 17), contributed to the study. Most participants were women (16), and their ages ranged from 22 to 57 years (Table 2). All participants received the training of HHCG and have no religious background. No participants withdrew from the study during the data collection and analysis period.

**Table 2:** Demographic characteristics of the participants (*n* = 17)

Characteristics		*N* (%)
**Sex**	Male	1(6)
	Female	16(94)
**Age (in years)**	18–30	5(29)
	31–40	5(29)
	41–50	5(29)
	≥ 51	2(12)
**Education**	Junior college	2(12)
	Bachelor’s degree	11(65)
	Master’s degree	4(23)
**Position**	Head nurse	5(29)
	Nurse	12(71)
**Marital status**	Unmarried	5(29)
	Married	12(71)
**Work experience (in years)**	≤ 5	3(18)
	6–10	7(41)
	≥ 11	7(41)
**Experience with the HHCG (in years)**	≤ 1	4(23)
	2–3	10(59)
	≥ 4	3(18)

**Fig. 1:**
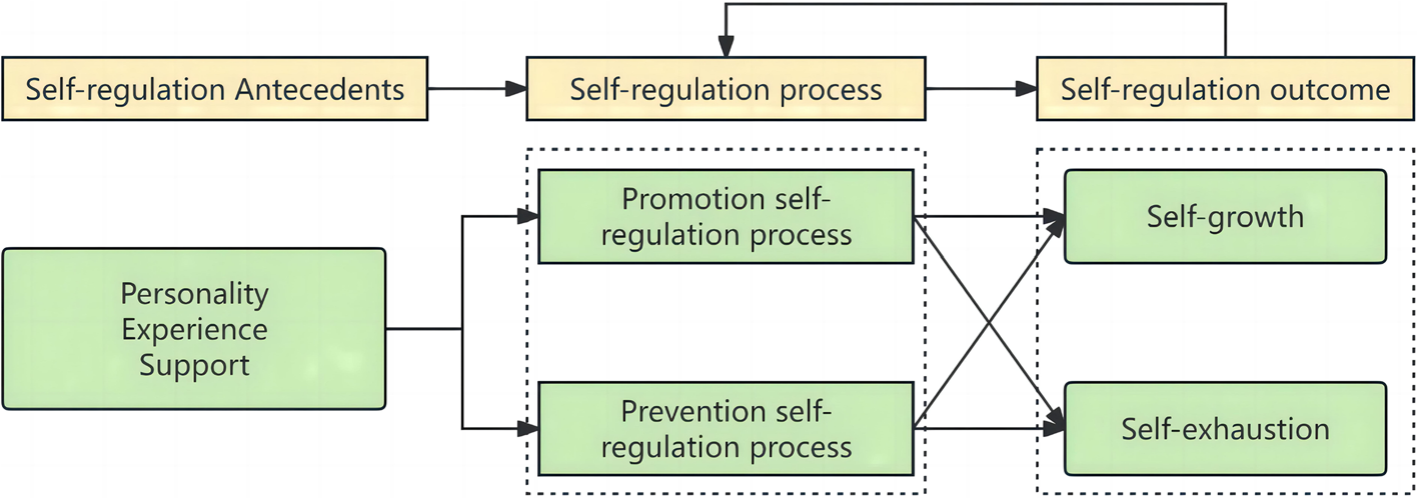
Theme structure

The study revealed three themes and seven subthemes (Table 3). Participants highlighted different antecedents of self-reflection, which influenced their tendencies towards self-regulation. When self-regulation was successful, participants experienced self-growth; in the converse case, self-exhaustion occurred (see Fig. 1 for the theme structure and Table 3 for the themes, subthemes and selected quotations from participants).

**Table 3:** Themes, subthemes and selected quotations from participant

Themes	Subthemes	Selected quotations from participants
Self-regulation antecedents include personality, experience, and support	The complex influence of personality tendencies	*I am a relatively introverted person. To be honest, I am not very good at comforting others. But I am very willing to become a good listener. (Participant 4, 53, female, married, works in a hospice unit, hosting HHCG approximately once every three months, a teacher of training for clinical nurse specialist in hospice care )*
	Life experience can affect nurses’ perception of HHCG	*I am also a psychological counsellor; I know some relaxation techniques and psychological counselling methods. I know how to detach myself from other people’s emotions, which is very helpful. (Participant 5, 36, female, married, works in a hospital, hosting HHCG approximately once a month, a third-level psychological counselor, a head nurse )*
	Perceived support is important for nurse self-regulation	*Sometimes, I go to our specialized psychological counsellor…Sometimes patients dump a lot of negative emotions on us. As ‘trash cans’ for them, we need to dump our emotions too. (Participant 12, 34, female, married, works in a hospital, hosting HHCG approximately once a month, )*
Promotion or prevention is a possible self-regulation process for nurses	Nurses believe that HHCG is meaningful for themselves and their patients	*They (patients) would tell me their demands; some wanted to go home, some didn’t want intubation, some wanted to go fishing. Some could be realized. I would try my best to help them, their family, who were also very grateful. (Participant 16, 32, female, unmarried, works in a hospital, hosting HHCG approximately once a week)*
	Nurses strive to improve their skills to cope with “troubles”	*We can use “pass on” instead of “death”, and we can express it tactfully… Also, if you feel that you cannot handle this issue well, you can temporarily skip discussing it. You can seek help from others, or we can handle it at a better time. The atmosphere of the end-of-life conversation can also be relaxed and warm. (Participant 4, 53, female, married, works in a hospice unit, hosting HHCG approximately once every three months, a teacher of training for clinical nurse specialist in hospice care)*
Both self-exhaustion and self-growth may be the outcomes of self-regulation	Be a better version of yourself	*I met a patient who always wanted a cemetery, but the family refused to take it seriously…I told the patient’s wishes to the family. After the patient left, the family member was very grateful to me and said “I feel that my dad [patient] had no regrets when he left; he was very peaceful. Thank you very much.” At that moment, I felt that my job was incredibly meaningful. (Participant 14, 57, female, married, works in a hospice unit, hosting HHCG approximately once every three months, a teacher of training for clinical nurse specialist in hospice care, a head nurse)*
	Nurses experience a cycle of negative emotions	*Sometimes family members can’t understand why we have end-of-life conversations with patients. They feel like we’re cursing their loved ones, and sometimes even patients: “I’m going to die? You come to talk to me about this.” I am really speechless; I am also very aggrieved, okay? (Participant 3,27, female, unmarried, works in a hospital, hosting HHCG approximately once a week )*

Abbreviation: *HHCG *Heart to Heart Cards Game

### Theme 1 self-regulation antecedents include personality, experience, and support

Self-regulation antecedents refer to the person-based variables that influence self-regulation and the corresponding behaviours. Our study identified three main antecedents, namely, personality tendencies, life experience and perceived support.

### Subtheme 1 the complex influence of personality tendencies

Many participants mentioned their personalities and highlighted the role of personality in end-of-life conversations. Personality affects the self-regulation process of participants, but our study did not reveal a clear relationship between these two factors. The participants indicated that introversion and extroversion did not affect the ways in which they engaged in end-of-life conversations. In contrast, personalities, whether extroverted or introverted, are influenced by the negative emotions generated by end-of-life conversations.

*I think that I am a sunny person, outgoing in daily life; I enjoy communicating with patients and having heart-to-heart talks, but I have done it (played the HHCG) twice recently; the overall atmosphere was quite heavy, and I feel a little down. I’m in a funk. I even feel repulsed. (Participant 7, female, unmarried, works in a hospital, hosting HHCG approximately once a week)*

### Subtheme 2 life experience can affect nurses’ perception of HHCG

Participants mentioned that their role identity, experience with end-of-life conversations, and reserves of relevant knowledge and abilities could affect their self-regulation methods. Rich knowledge could help them adjust their own state more effectively, but similar experiences could also expose them to trauma or lead them to develop a high level of empathy.

*After having more experience (with the HHCG), I am not as easily influenced by [the patient]. It does not mean that I become more indifferent but [that I become] more rational… I often tell myself that those things belong to him [the patient], not to me. (Participant 1, 29, female, unmarried, works in a hospital, hosting HHCG approximately once a week)*

*When patients talk about their children, I quickly empathize with them. I also have a 2-year-old daughter; I can’t imagine my daughter losing me… I can’t help but cry too. (Participant 8)*

### Subtheme 3 perceived support is important for nurse self-regulation

All participants identified psychological support as very important and reported that powerful support could help them overcome negative emotions and adjust their mindsets more effectively. The support mentioned by the participants was provided mainly by their partners, who also played the HHCG with patients, as well as professional psychological counsellors.

*I don’t want to bring negative emotions home. I communicate with my colleagues; sometimes she comforts me, sometimes I comfort her, and I think that without her, I might not be able to persist. (Participant 9, 28, female, unmarried, works in a hospital, hosting HHCG approximately once a week)*

### Theme 2 Promotion or prevention is a possible self-regulation process for nurses

We identified two self-regulation processes based on participants’ expressions: one process involved a primary focus on obtaining benefits, and the other involved a primary focus on preventing danger. It is notable that these two self-regulation processes are not identified as good or bad. The same person may employ different self-regulation processes to respond to different situations. In other words, a person can undergo two self-regulation processes.

### Subtheme 1 nurses believe that HHCG is meaningful for themselves and their patients

Some participants noted that they believed that end-of-life conversations are necessary and meaningful; although some setbacks may occur during the conversation, continuing such conversations is worthwhile.

*She [the patient] told me, ‘After talking to you, I suddenly feel that I am not so uncomfortable.’ I feel that it is meaningful for me to do these things and what I am needed to do. “I think I can help more people through this game, which may be difficult. But, no trouble can cause my heart to waver! I will keep it up. (Participant 10, 45, female, married, works in a hospital, hosting HHCG with variable frequency, a head nurse)*

### Subtheme 2 nurses strive to improve their skills to cope with “troubles”

A small number of participants noted that although they engaged in the HHCG with advanced cancer patients in this situation with the goal of completing tasks assigned by their superiors, they also strove to learn communication skills and avoid causing resentment or misunderstandings among patients, taking into account their own responsibilities. Some participants also indicated that when they perceived that the topic may have caused emotional discomfort to the other party and they may not have been able to handle this situation, they found it necessary to skip the topic in question.

*I don’t really want to play this Heart to Heart Cards Game because, you know, it’s not easy for us to discuss the topic of “death” with these patients. But the head nurse asked me to do it and said that it’s very meaningful. Well, it’s okay, and it made me a little group leader. So, I have to learn how to do it; at least, I need to know how to deal with the … avoid making patients think that I’m cursing them. (Participant 3, 27, female, unmarried, works in a hospital, hosting HHCG approximately once a week)*

### Theme 3 both self-exhaustion and self-growth may be the outcomes of self-regulation

All participants indicated that engaging in end-of-life conversations with advanced cancer patients had impacts on them, but they reported almost polarized opinions concerning performance. Some participants indicated that they had obtained many gains and achieved self-growth. Another group of participants believed that they could not regulate their mentality well, thus resulting in self-doubt and a sense of powerlessness.

### Subtheme 1 be a better version of yourself

Participants noted that many patients and their families expressed gratitude to them, thus causing them to feel that they have realized their professional value. In addition, some participants claimed that they had learned a great deal from patients, that they had become more outstanding versions of themselves and that they were more grateful for their current lives.

*Through the patient’s affairs, sometimes I do self-reflection. I feel that I am very lucky. These thoughts will unknowingly affect me… In short, I cherish and enjoy my current life more… Nurses have to learn to love themselves before they can love others. (Participant 5, 36, female, married, works in a hospital, hosting HHCG approximately once a month, a third-level psychological counselor, a head nurse)*

*There must be times when I’m feeling low; then, I play basketball and the piano, and these make me feel peaceful… I feel like I am a very strong and powerful person now. Perhaps, after having listened to so many stories, I feel that my mind has broadened a lot. I think I like current myself. (Participant 6,49, female, married, works in a hospital, hosting HHCG with variable frequency, a head nurse)*

### Subtheme 2 nurses experience a cycle of negative emotions

Some participants expressed their unwillingness to continue playing the HHCG. Patients displayed many negative emotions or made demands. When patients could not handle their negative emotions or when their simple demands could not be met, the participants felt that what they were doing seemed to be meaningless, thus leading to a sense of self-doubt and powerlessness. Sometimes, participants’ kindness could even be misunderstood, causing them to feel aggrieved.

*I doubted myself even more. During that time, I was also very depressed. I felt that I was full of negative energy. Many times, I felt like I couldn’t hold it anymore. I wondered if I was not suitable for this. In fact, I am a super resistant-to-negative-energy person. It feels so difficult. It just happens that this job involves doing this. Sometimes I feel like they are consuming me. (Participant 11, 41, male, married, works in a hospice unit, hosting HHCG approximately once a month)*

*Sometimes I feel powerless. Advance care planning is not perfect; lots of (patients’) rights cannot be guaranteed. I can do little. (Participant 2, 39, male, married, works in a hospice unit, hosting HHCG with variable frequency)*

## Discussion

This study explored the experiences of and self-regulation exhibited by nurses in the context of in-depth end-of-life conversations with advanced cancer patients. This type of qualitative study can provide in-depth, data-rich contextual information that can guide nursing management and specialist nursing.

The first theme included several factors that can affect the self-regulation process; we identified these factors as self-regulation antecedents. Among the factors pertaining to the development of self-regulation that we studied, personality indicators play an important role [19, 33]. A meta-analysis [33] revealed that higher levels of extraversion, openness, agreeableness, and conscientiousness are associated with the more frequent use of typically adaptive emotion regulation strategies (problem solving) and the less frequent use of typically maladaptive emotion regulation strategies (avoidance). However, it is worth noting that personality type predicts self-regulation tendencies rather than regulating strategies. Our study revealed that some introverted individuals actively seek ways of engaging in end-of-life conversations with patients with the goal of helping them, whereas outgoing participants may also choose to avoid negative emotions. Further quantitative research may be needed to explore the relationship between personality types and self-regulation strategies.

Alongside personality, self-regulation processes are significantly influenced by knowledge and experiences, which accumulate as a result of nurses’ interactions with other patients, colleagues, families, and the environment as a whole. A previous study demonstrated that self-regulation is closely related to cultural background and knowledge [34]. Our study further revealed that nurses are more likely to exhibit strong empathy and to become trapped in a vortex of negative emotions if they share similarities with patients during end-of-life conversations. However, this “resonance point” is invisible and cannot be detected even by the nurses themselves before engaging in the end-of-life conversation. Given that these self-regulation antecedents are almost unmodifiable and that some factors are difficult to identify, we must equip nurses with additional coping and communication skills. As the participants noted, “After engaging in end-of-life conversations with more patients, they also became more composed.” Experienced nurses can share their experiences and help “green” nurses improve their skills in end-of-life conversation [6].

Interestingly, we found that almost all participants expressed their reluctance to transmit negative emotions to their families, which resulted in their inability to seek support from their families actively. Participants were more inclined to receive help from colleagues and professional psychologists. The intention of nurses to seek support required them to pay attention to the mental health of department staff and provide them with channels for psychological counselling [35]. Additionally, nurses could establish a team to provide opportunities for their negative emotions to reach catharsis, and they could establish a platform for peer support [36].

The second theme focused on two types of self-regulation processes that can be summarized in terms of participant expression. Some participants firmly believed that engaging in end-of-life conversations with advanced cancer patients could reduce their regret and improve their quality of life. Despite the difficult and long road they faced, they were still willing to perform this task. These participants were associated with promoting self-regulation, and their behaviour was driven primarily by their consideration of benefits. Another type of self-regulation process is prevention self-regulation. This type does not entail that nurses refuse to engage in or evade such conversations; rather, they choose to avoid certain topics to avoid foreseeable “conflicts” or “out of control emotions”.

Our participants noted that end-of-life conversations can help nurses understand the patient’s wishes and provide better care; accordingly, it is precisely because they perceive the benefits of end-of-life conversations for advanced cancer patients who they are motivated to do so. These findings are in line with the growing body of evidence in this field and contribute additional insights concerning experiences of nursing. End-of-life conversations are considered to constitute core component of interactions among patients, families, and health care staff [37]. Such conversations offer the opportunity for patients to express their experiences and thoughts; prepare for their departure; share their decisions; and contemplate the meaning of life [38]. A structural equation model revealed that encouragement and rewards have significant effects on employee performance [39]. Incentive policies (such as praising nurses or conveying patients’ gratitude to nurses) may be more useful for nurses who exhibit promotion self-regulation.

Cultural misconceptions and biases towards dying and death among Chinese people, in general, have continued to be identified as common, and end-of-life education in mainland China has not received sufficient attention [40]. Many nurses have not received systematic training in end-of-life care and thus lack the skills necessary to deal with emotional loss or complaints from patients [40, 41]. It is challenging and stressful for Chinese nurses, especially green hand nurses, to engage in end-of-life conversations with patients [41]; therefore, it is understandable that these nurses want to avoid discussing the topic of death. Our interviews revealed that many participants were worried that end-of-life conversations would be misunderstood by patients as conveying the suggestion that “I’m about to die”, which could lead to unnecessary misunderstandings. We maintain that nurses who exhibited prevention self-regulation tendencies should be provided with additional guidance and training, such as in ways of using language arts to avoid misunderstandings on the part of patients, coping strategies to address unexpected situations, and ways of skipping topics when they anticipate conflicts.

The final theme focused on the fact that self-regulation success leads to self-growth, whereas failure leads to self-exhaustion. The success or failure of self-regulation is not determined by the type of self-regulation in question but rather by whether the initial motivation for self-regulation is satisfied. Promoting self-regulation may lead to self-exhaustion if sufficient benefits are not perceived as having been obtained, while participants who exhibit protective self-regulation may experience self-regulation failure if they do not avoid danger.

A qualitative study revealed misunderstandings among patients regarding end-of-life care, which can lead to difficulties in the context of end-of-life conversations between nurses and patients [42]. Our participants noted that being misunderstood or even blamed by patients for their kindness could cause them to feel very aggrieved. In addition to strengthening the training of nurses in conversation skills, creating a cultural background that features a rational view of death and a harmonious and empathetic environmental atmosphere may be more important [42–44].

In addition, when participants are determined to help patients but are unable to fulfil their wishes for various reasons (such as a lack of support from family members or incomplete advance care planning policies) or when the support they receive is very limited, they may experience a deep sense of powerlessness. Some participants could reconcile with themselves through self-regulation, while others gradually developed self-doubt and even experienced thoughts of resignation. Nurses’ self-doubt and powerlessness can easily lead to burnout and impair their ability to exhibit attuned communication and empathy, thereby affecting patient care and satisfaction [45]. If nursing managers do not pay attention to the mental health of the people who provide care, then the provision of exceptional patient-centred, high-quality health care is certain to suffer [45]. Mindfulness-based interventions have been demonstrated to be able to increase participants’ ability to withstand sustained exposure to unpleasant/pleasant dynamics [46]. Nursing managers can consider organizing psychological workshops, and nurses can actively learn psychological relaxation techniques.

Nurses can not only develop a sense of professional identity while helping patients but also expand their thinking and increase inclusivity. One study [47] revealed that a common result of nurses’ use of spiritual care to provide comfort to patients is the achievement of peace by both the patient and the nurse, thus indicating that nurses can reciprocally receive strength from patients through these interactions. Some participants mentioned that music and exercise are good ways in which they can regulate themselves. Organizational managers can provide opportunities for experienced nurses to share good self-regulation methods. Future research can also explore the factors that promote and hinder the success of self-regulation and develop corresponding interventions.

The findings of our research suggest that self-regulation has significant impacts on the outcomes of nurses. Peer support, professional psychological counselling, and training in self-regulation skills are essential for people participating end-of-life conversations. The results of this study can contribute to the development of end-of-life conversation training approaches and practices by facilitating successful self-regulation among nurses. Although doing so was not the focus of this study, our study also offers evidence that can highlight the significance of end-of-life conversations with cancer patients.

This study has several limitations. First, the study sample was recruited only from major hospitals in three cities. We tried our best to select participants from different institutions, with different demographic characteristics, and with different levels of work experiences to ensure the diversity of the sample. Second, due to limitations pertaining to objective factors, most of our participants were women, which may have led to some degree of bias. Future research can focus on the views of male nurses. Third, We did not explore the differences in nurses’ understanding of the term “self-regulation”, although no nurses asked us for further explanation, which may cause some bias. Besides, the findings of this research may not be generalizable to other cultural backgrounds, such as Western cultures. However, most of the findings are supported by the literature, which makes the results applicable.

Given the importance of self-regulation centrality to our understanding of nurses’ psychology and behaviour, this study used a qualitative approach to explore the self-regulation of nurses in the context of end-of-life conversations with advanced cancer patients. Based on the results of this study, university and hospital managers or educators should develop systematic education for end-of-life clinical practice and promote public awareness of advanced care planning to ensure that end-of-life conversations can be conducted more easily. Furthermore, empirical research is needed to explore appropriate strategies and interventions to provide support to health care personnel with different self-regulation styles.

## Conclusion

This qualitative study enhances our knowledge of nurses’ self-regulation both during and after end-of-life conversations with advanced cancer patients. We found that nurses generally believe that end-of-life conversations are worthy of clinical promotion since they can help advanced cancer patients receive improved quality of care. However, although some nurses reported that they had achieved self-growth through successful self-regulation, we cannot ignore the fact that some nurses had already developed serious negative resistance in emotional terms. Nursing managers must provide nurses with appropriate guidance based on the self-regulation process and offer them professional psychological assistance to the greatest extent possible.

## Data Availability

The full interviews’ data are not publicly available due to privacy or ethical restrictions. However data is available upon reasonable request from the first author.

## Abbreviation

HHCG: Heart to Heart Cards Game
